# Influence on emergency digit replantation and outcome assessment after COVID-19 virus nucleic acid testing normalization

**DOI:** 10.3389/fsurg.2022.1078933

**Published:** 2023-01-06

**Authors:** Kunqi Zhang, Rui Zhang, Shanyu Li, Shenghe Liu, Feiyan Wang, Jia Xu, Qinglin Kang

**Affiliations:** Department of Orthopedics, Shanghai Sixth People's Hospital Affiliated to Shanghai Jiao Tong University School of Medicine, Shanghai, China

**Keywords:** COVID-19, nucleic acid test, digit replantation, outcome assessment, normalization, policy

## Abstract

**Objective:**

The study aims to compare the implementation and prognosis of emergency digit replantation surgery before and after normalized corona virus disease 2019 (COVID-19) nucleic acid testing for patients taking emergency operation and to explore the influence of normalized COVID-19 nucleic acid testing on replantation surgery.

**Method:**

Normalized COVID-19 nucleic acid testing for patients taking emergency operation has been carried out since 1 August 2021 at our hospital, which means each patient who needs emergency surgical treatment has to obtain either positive or negative results of COVID-19 nucleic acid before entering the operating room. This research reviewed and compared the prognosis of the injured extremity that had emergency severed digit replantation between June and September 2021, at the Shanghai Sixth People's Hospital Affiliated to Shanghai Jiao Tong University School of Medicine, and analyzed the impact of normalized COVID-19 nucleic acid testing on the outcome of the replanted fingers of different severity using disability of arm-shoulder-hand (DASH) and hand injury severity scoring (HISS) scoring systems.

**Results:**

A total of 54 cases with 74 severed replanted phalanges were included replanted by the research group between 1 August and 30 September 2021, without any COVID-19 suspected/confirmed case detected. Compared with previous period (1 June to 31 July, 2021), although the interval between emergency visits and emergency replantation did increase significantly after normalized COVID-19 nucleic acid testing [(3.83 ± 0.94) to (1.77 ± 0.67) h, *P *< 0.05], we observed no significant difference in the improvement rate of the DASH scoring of the disabled upper extremity 3-month postoperatively (*P *= 0.538) nor in the complication rate (*P *= 0.344). Moreover, there was no significant difference in the improvement rate of the DASH scoring of the disabled upper extremity 3-month postoperatively in patients with different traumatic severities before and after normalized COVID-19 nucleic acid testing (moderate *P* = 0.269, severe *P* = 0.055, major *P* = 0.149).

**Conclusion:**

Despite the preoperative delay, the policy of COVID-19 nucleic acid testing normalization does not have explicit influence on the short-term outcomes of emergency digit replantation surgery. With this evidence, microsurgeons could pay attention to the patients' anxiety and spend more effort in comforting them during the prolonged preoperative wait. These insights may have implications for other emergency department resource management whenever a social crisis occurs.

## Introduction

Prevention and control of corona virus disease 2019 (COVID-19) pandemic has attracted worldwide concern since 2020, and most countries and regions have established temporary social distancing policies during waves of the pneumonia outbreak (1). As one of the first batch of countries identifying COVID-19 pandemic, China has quickly controlled social transmission of the virus in the first wave of pandemic for the recovery of society and economy, thanks to the precise and strict policy implementation of Chinese public health system (2). Powerful molecular diagnostic methods have been explored to detect the infected, among which nucleic acid detection of severe acute respiratory syndrome coronavirus 2 (SARS-COV-2) has gained an indispensable role due to its simplicity and fast report (3).

The COVID-19 pandemic and related policies have also shed impact on the frequency and disease distribution of emergency department visit because of the geographic restriction and changes in daily activities (4). As for hand trauma, although the incidence of emergency visit decreased globally in the first wave of the pandemic, the rate of emergency operation was reported unchanged and eventually the incidence returned to normal and remained unaffected by the following pandemic waves (5–8). Replantation of severed digits represents a hallmark in hand surgery, and survival rate of the digits has been increasing thanks to advances in microsurgery (9). Meanwhile, the rate of emergency digit replantation was reported to be relatively constant and even higher during waves of pandemic (4, 5, 10).

In addition to the Chinese dynamic zero-COVID policy, hospitals in China have also addressed preoperative guidelines to protect staff and patients against SARS-COV-2. Since 1August 2021, the policy of COVID-19 virus nucleic acid testing normalization has been established, according to which patient with non-life-threatening indications for emergency surgery should obtain the result of the virus nucleic acid detection preoperatively to prevent iatrogenic transmission. Although the ischemic time has been shown not to be significantly associated with survival rate and long-term outcomes, digit replantation surgery is still considered emergent by patients and their relatives due to cultural and religious reasons (11, 12). Despite the improvement in virus nucleic acid detection, it still takes hours before the report of the detection, which prolongs the time interval from emergency department visit to surgery compared with only receiving typical preoperative laboratorial and radiological examinations (3). Unfortunately, little evidence has demonstrated the relationship between the prolonged time interval and outcomes of emergency digit replantation during this special period.

This study aimed (1) to compare the implementation and prognosis of emergency digit replantation surgery before and after normalized COVID-19 nucleic acid testing for patients taking emergency operation and (2) to explore the influence of normalized COVID-19 nucleic acid testing on the replantation surgery.

## Patients and methods

### Ethical approval and inclusion and exclusion criteria

This is a single-centered prospective study that included data from patients (group N) who received emergency phalanges replantation due to digit severed lesions from 1 August to 30 September 2021 at the Shanghai Sixth People's Hospital Affiliated to Shanghai Jiao Tong University School of Medicine. Data from patients who received emergency phalanges replantation from 1 June to 31 July 2021 were recorded as a control group (group C).

Patients who met the following status were included: (1) single-planed open amputated digit lesion without previous phalangeal replantation history and with one or more digits involved; (2) either complete or incomplete amputation that caused complete or incomplete loss of blood supply of the distal part; (3) digit replantation accepted by the patient or the patient's guardian; (4) combined with other injuries, but the rest of the injuries in both upper extremities did not require emergency amputation surgery.

Patients who met the following status were excluded: (1) amputated digits were caused by hot crush violence or with degloving injury; (2) intolerance of surgery due to systematic diseases, such as severe cardiovascular diseases, epilepsy, intoxication, and drug allergy; (3) digit replantation was rejected by the patients.

The study was in accordance with the 1975 Declaration of Helsinki. Ethical approval of the study was obtained from the Institutional Ethics Committee of Shanghai Sixth People's Hospital [2021-KY-113(K.)] The patients and/or their bailors were informed that data from the case would be submitted for publication and their consent was obtained.

### Management protocols for emergency digit amputation injuries before and after COVID-19 virus nucleic acid testing normalization

Patients with digit amputation injuries first received basic COVID-19 screening according to their history of epidemiology and axillary temperature at emergency previewing counter. Patients were sorted as preclusion-required patients after first screening (PRFS) and were guided to the fever clinic (FC) through a restricted lane for further COVID-19 virus nucleic acid test, blood routine examination, and pulmonary computed tomography (CT) in order to preclude COVID-19 if they met one of these situations: (1) an axillary temperature above 37.3°C, (2) a sojourn history in moderate- or high-risk regions for COVID-19 pandemic countrywide or abroad within 14 days, (3) close contact with COVID-19 patients, and (4) work history at ports. Otherwise, patients negative for COVID-19 after first screening (NFS) were guided toward the emergency room for traumatic orthopedics and received surgery- and anesthesia-related examinations as well as COVID-19 virus nucleic acid test, blood routine examination, and pulmonary CT. Diagnostic criteria of COVID-19, including history of epidemiology, and laboratory and radiological examinations, were in accordance with the National COVID-19 diagnosis and treatment guidelines (Interim version 8) (13). All patients were required for routine follow-up at 1, 2, 4, 6, 8, and 12 weeks postoperatively, and clinical outcomes were evaluated at each follow-up.

**Figure 1:**
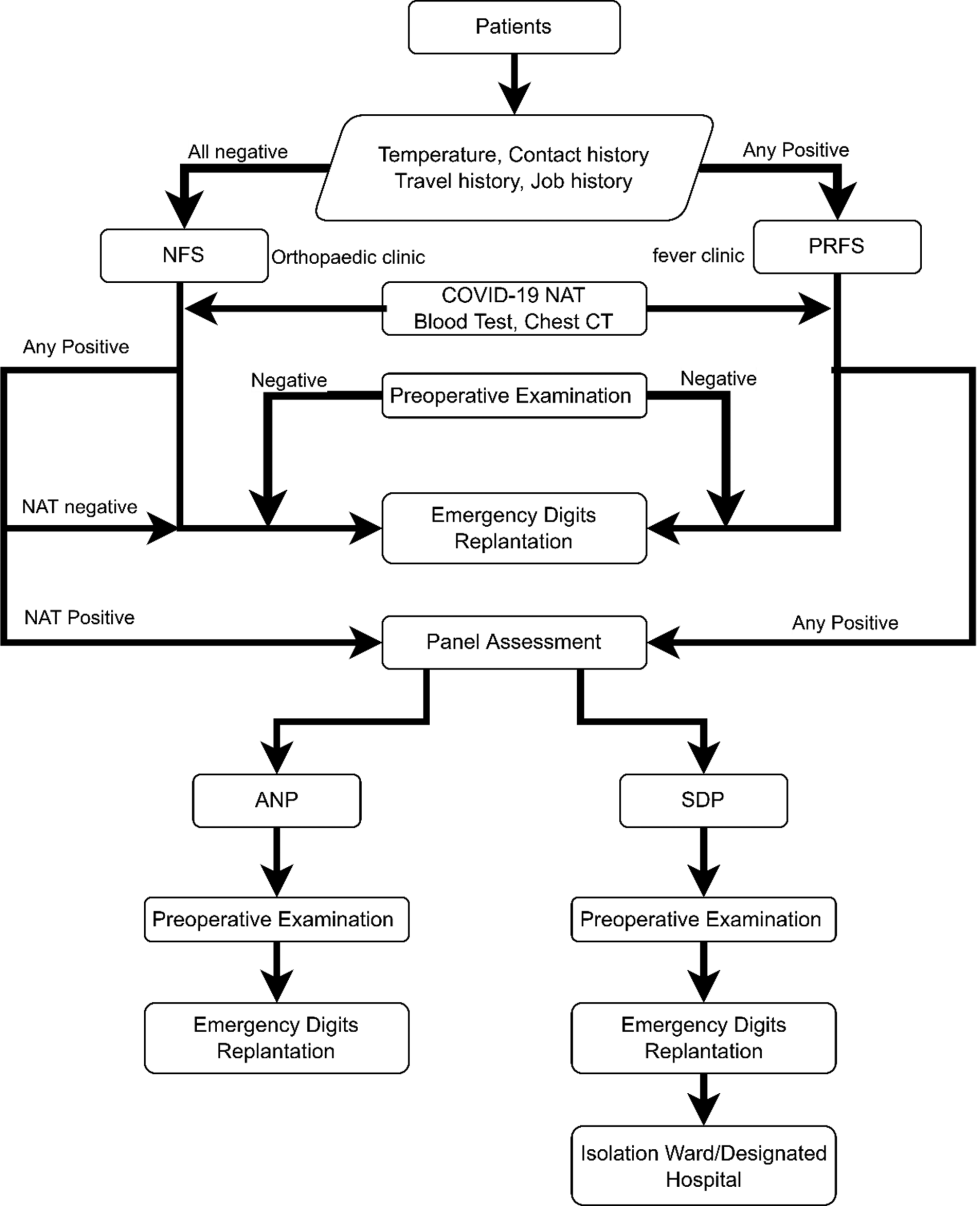
Diagnosis and management process for emergency severed extremity replantation before COVID-19 virus nucleic acid testing normalization. COVID-19, corona virus disease 2019.

Before 1 August 2021, NFS patients with negative results of blood routine examination and pulmonary CT, and PRFS patients with negative results of COVID-19 virus nucleic acid test, blood routine examination, and pulmonary CT could receive phalanges replantation surgery under brachial plexus anesthesia in a negative-pressure operating room once they finished surgery- and anesthesia-related examinations and were then transported to special units for amputation injury postoperatively. NFS patients with positive results of either blood routine examination or pulmonary CT and PRFS patients with at least one positive result of COVID-19 virus nucleic acid test, blood routine examination, and pulmonary CT were sorted as suspect patients after first screening (SFS), and further assessment by the epidemiology prevention and control expert group was required to distinguish assessed negative patients (ANP) and COVID-19 suspected/diagnosed patients (SDP). ANP could receive replantation surgery under regular protocols in a negative-pressure operating room after preoperative examinations and were then transported to special units for amputation injury postoperatively. Twenty-four hours after the operation, another COVID-19 virus nucleic acid test was performed, and patients with positive result were transferred to specified hospitals for COVID-19 patients. SDPs were guided to the negative-pressure operating room for replantation surgery through a restricted lane after finishing preoperative examinations, and thorough sterilization and a 90-min lockdown of the operating room were acquired postoperatively. SDPs were temporarily transported to isolated wards and were then transferred to specified hospitals for COVID-19 patients postoperatively (Figure 1).

Since 1 August 2021, results of COVID-19 virus nucleic acid test within 72 h should be compulsorily obtained before emergency replantation surgery at our hospital, namely, COVID-19 virus nucleic acid testing normalization. NFS patients could only receive operation after obtaining negative results of COVID-19 virus nucleic acid test, blood routine examination, and pulmonary CT in the negative-pressure operating room. NFS or PRFS patients with at least one positive result require further assessment by the epidemiology prevention and control expert group before arranging for the operation. Except for SDPs, additional COVID-19 virus nucleic acid test, blood routine examination, and pulmonary CT were arranged for patients with fever due to unknown cause. This period is the so-called COVID-19 virus nucleic acid testing normalization.

**Figure 2:**
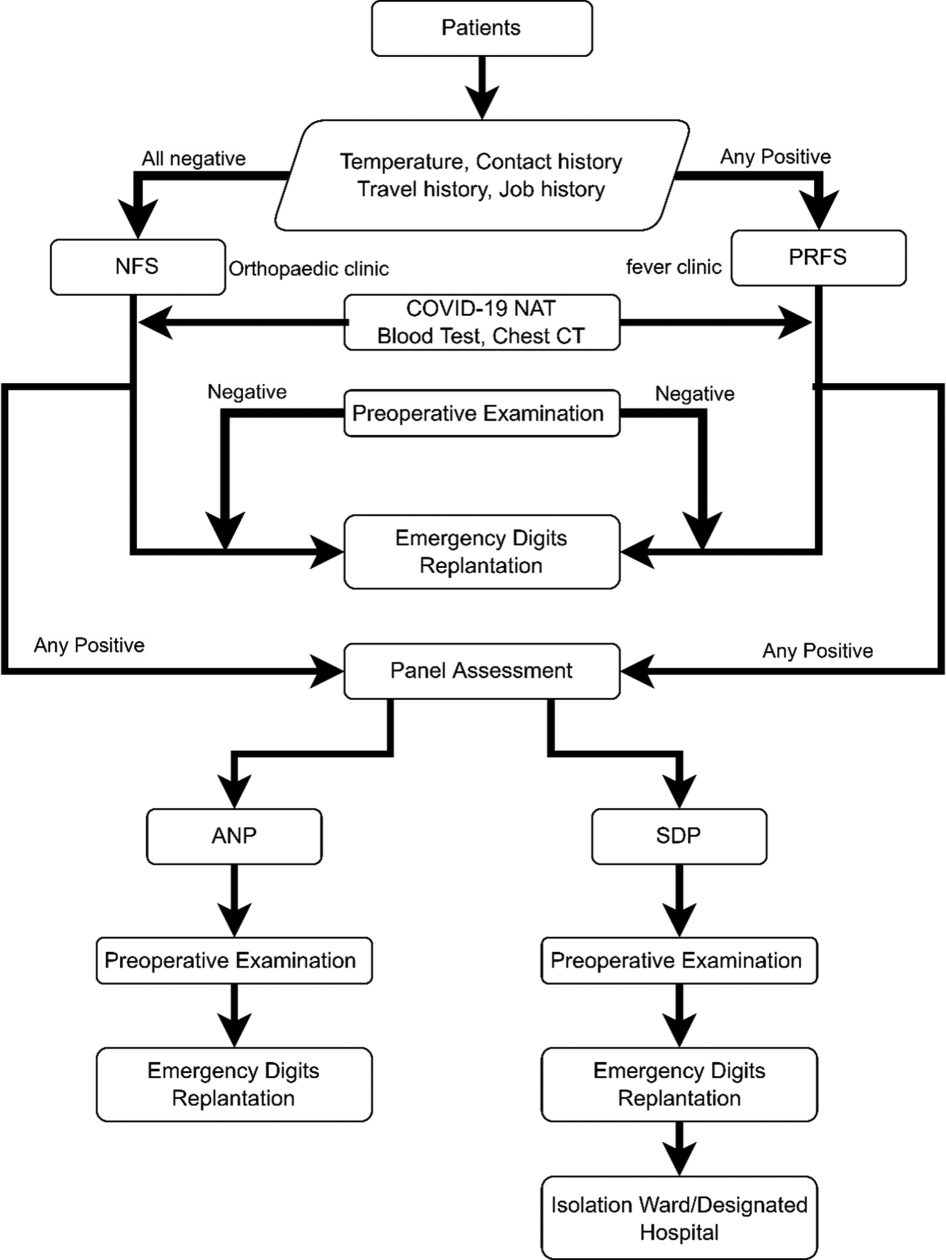
Diagnosis and management process for emergency severed extremity replantation after COVID-19 virus nucleic acid testing normalization. COVID-19, corona virus disease 2019.

Emergency digit replantation was performed by the same surgical team under brachial plexus anesthesia. Main structures of each amputated digit, including bilateral neurovascular bundles, tendons, adequate veins, and bone, were cautiously reconstructed. Three veins and volar bilateral arteries were anastomosed in routine. Standard second-level protection, same as protection of usual hand surgery, was applied for surgical team when performing operation for non-SDPs. Standard third-level protection, with additional protective suit under normal surgical gown, two extra pairs of sterile gloves, and a face shield, was applied for surgical team when performing operating on SDPs (13, 14) (Figure 2). Postoperative care equaled to the routine managements in a published article (15).

### Data collection

Demographic data, history of epidemiology, physical examination results, experimental results, treatment, and prognosis data were collected through electronic medical record system. These data included gender, age, patients epidemiological risk degree, mechanisms of injury, number of affected digits, names of affected digits, lacerated and repaired structures of affected digits, time from injury to surgery (TIS), time from emergency visit to surgery (TVS), operation time (OT), hospitalization time (HT), complications, and clinical outcomes. Hand injury severity scoring (HISS) was applied for the assessment and grading of the severity of the injury, which was performed by assigning scores for the lesions in the aspect of skin, nail, nerve, intrinsic muscle and tendon, bone, and ligament of the hand and calculating the weighing sum according to the status of importance of the amputated digit (16). Disability of arm-shoulder-hand (DASH) questionnaire was applied both preoperatively and 3 months after the operation to evaluate the improvement of the affected upper extremities, which includes two major parts regarding the ability of upper extremity activities (Part A) and severity of symptoms (Part B) (17). Clinical grading was performed by two separate researchers, and a third evaluation was done if the first two grading varied.

Grading of HISS for severity of the injury: minor (0–20 points), moderate (21–50 points), severe (51–100 points), and major (above 100 points).

DASH score was calculated as follows:

$$\mathrm{DASH}=\frac{A_{\mathrm{sum}}+B_{\mathrm{sum}}-30}{1.20}$$

We introduce a novel parameter, the improved rate of DASH score (impDASH), to compare the outcomes of emergency digit replantation before (group C) and after (group N) COVID-19 virus nucleic acid testing normalization, calculated as follows:

$$\mathrm{impDASH}=\frac{\mathrm{DAS}H_{\;\mathrm{preoperative}}-\mathrm{DAS}H_{\mathrm{three}-\mathrm{month}}}{\mathrm{DAS}H_{\;\mathrm{preoperative}}}\times 100\%$$

### Statistical analysis

Statistical analysis was performed using GraphPad Prism 5 (GraphPad software, CA, United States). Normal distribution was checked statistically. Descriptive statistics were expressed as mean ± standard deviation (`*x ± s*) for continuous numerical variables and as number and percentage for categorical variables. Unpaired two-group *t*-test was applied to compare age, time related with management and follow-up, numbers of injured structures, and objective outcomes (the DASH score). Chi-square *t*-test was applied to compare characteristics of injuries of the two groups. *P* value < 0.05 was considered a significant difference.

## Results

### Basic information and overall outcomes

Fifty-eight patients who received emergency digit replantation were included in group C from 1 June to 31 July 2021. None of them were assessed as SDPs, and two patients (3.4%) were sorted as PRFS or SFS patients. Mean age of the patients in group C was 40.4 ± 13.5 years (4–72 years). Mean TVS was 1.77 ± 0.67 h. Eighty-seven severed digits were replanted, with 81 survival fingers (93.1%), average HISS of 107.8 ± 79.0 points, and average preoperative DASH score of 53.8 ± 17.6 points. The severity of the injury was graded as 19 moderate, 17 severe, and 22 major in group C according to HISS of the patients.

Fifty-four patients who received emergency digit replantation were included in group N from 1 August to 30 September 2021. Five patients were evaluated as PRFS patients, and another patient (1.9%) was tested as COVID-19 suspected patient; they all were assessed as an ANP by the epidemiology prevention and control expert group for emergency replantation surgery. Mean age of the patients in group N was 46.4 ± 16.3 years (2–81 years). Mean TVS was 3.83 ± 0.94 h. Seventy-four severed digits were replanted, with 72 survival fingers (97.3%), average HISS 89.4 ± 51.6 points, and average preoperative DASH score 54.3 ± 16.7 points (Table 1). Severity of the injury was graded as 15 moderate, 18 severe, and 20 major in group N according to HISS of the patients.

**Table 1:** Comparison of emergency finger replantation before and after COVID-19 virus nucleic acid testing normalization.

Surgery information	Before virus nucleic acid testing normalization (2021, 6–7)	After virus nucleic acid testing normalization (2021, 8–9)	*t* value/*χ*^2^ value	*P* value
Surgery numbers	58	54		
Fingers	87	74		
Male [case (%)]	49 (84.5)	43 (79.6)	0.449	0.503
Age (year, `*x* ± *s*)	40.4 ± 13.5	46.4 ± 16.3	2.118	0.037*
Screening grading [case (%)]				
NFS	56 (96.6)	48 (88.9)	2.476	0.116
PRFS	2 (3.4)	5 (9.3)	1.612	0.204
SFS	0	1 (1.9)	1.084	0.298
ANP	2 (3.4)	6 (11.1)	2.476	0.116
SDP	0	0	—	—
Injury mechanism [case (%)]			0.342	0.559
Incision	47 (81.0)	46 (85.2)		
Crush/	11 (19.0)	8 (14.8)		
Replantation finger (left/right/total) (%)			1.938	0.747
Thumb	13/7/20 (23.0)	17/5/22 (29.7)		
Index	15/6/21 (24.1)	3/9/12 (16.2)		
Middle	9/7/16 (18.4)	6/8/14 (18.9)		
Ring	11/6/17 (19.5)	5/10/15 (20.3)		
Little	8/5/13 (14.9)	5/6/11 (14.9)		
Preoperative score (*x* ± *s*)				
HISS	107.8 ± 79.0	89.4 ± 51.6	1.437	0.154
DASH	53.8 ± 17.6	54.3 ± 16.7	0.138	0.890
TIS (h, *x* ± *s*)	4.59 ± 1.08	5.97 ± 1.06	6.741	<0.0001*
TVS (h, *x* ± *s*)	1.77 ± 0.67	3.83 ± 0.94	13.36	<0.0001*
OT (h, `*x* ± *s*)	1.38 ± 0.86	1.43 ± 0.59	0.329	0.743
HT (day, *x* ± *s*)	4.3 ± 0.7	3.9 ± 0.9	2.160	0.033*
Survival fingers	81 (93.1%)	72 (97.3%)	1.489	0.222
Postoperative complications [fingers (%)]	27 (31.3)	18 (24.3)	0.894	0.344
Infection	2 (2.3)	2 (2.7)	0.027	0.870
Bleeding	0	0	—	—
Distal necrosis	6 (6.9)	1 (1.4)	2.957	0.086
Delayed union/nonunion	2 (2.3)	2 (2.7)	0.027	0.870
Joint mobility disorder	13 (14.9)	5 (6.8)	2.698	0.101
Tendon rupture/adhesion	8 (9.2)	3 (4.1)	1.661	0.198
Joint degeneration/fusion	5 (5.7)	2 (2.7)	0.891	0.345
Peripheral sensory disorder	4 (4.6)	8 (10.8)	2.238	0.135
Other	0	0	—	—
Postoperative DASH (`*x* ± *s*)	13.1 ± 12.5	13.5 ± 8.9	0.190	0.850
DASH augment [`*x* ± *s* (%)]	78.2 ± 16.1	76.4 ± 14.4	0.618	0.538

COVID-19, corona virus disease 2019; NFS, patients negative for COVID-19 after first screening; PRFS, preclusion-required patients after first screening; SFS, suspect patients after first screening; ANP, assessed negative patients; SDP, COVID-19 suspected/diagnosed patients; HISS, hand injury severity scoring; DASH, disability of arm-shoulder-hand; TIS, time from injury to surgery; TVS, time from emergency visit to surgery; OT, operation time; HT, hospital time.

*P value <0.05.

It is obvious that results of COVID-19 virus nucleic acid test from only 3.4% patients in group C were required before receiving emergency digit replantation, while results of COVID-19 virus nucleic acid test from all patients were required in group N.

### Comparisons of TVS and outcome of the replantation surgery before and after COVID-19 virus nucleic acid testing normalization

Although patients in group N had an older age (46.4 ± 16.3 years) compared with patients in group C (40.4 ± 13.5 years), there remained no significant difference (95% CI: −1.1 to 12.6, *P *= 0.100). Mean TVS was significantly prolonged in group N (3.83 ± 0.94 h) compared with that of group C (1.77 ± 0.67 h) (95% CI: 1.8 to 2.4, *P *< 0.05). Before COVID-19 virus nucleic acid testing normalization, a total of six fingers did not survive (ring and litter finger in a patient received 2–5 left fingers replantation HISS 217, and 1–4 necrosis received 1–5 left fingers replantation, HISS 164), while two fingers after the policy (left thumb received left thumb replantation HISS 42, and left index finger received left index finger replantation HISS 36). There was no significant difference (*P* = 0.222) in survival rate before and after the policy.

Lower HISS was also observed in group N (89.4 ± 51.6 points) compared with that of group C (107.8 ± 79.0 points), but there was still no significant difference (95% CI: −7.0 to 43.9, *P *= 0.154). Although DASH scores of patients in both groups were significantly improved (*P *< 0.05), no significant difference was observed in the aspects of preoperative DASH score (*P *= 0.890), DASH score 3 months after operation (*P *= 0.850), impDASH (95% CI: −4.0 to 7.6, *P *= 0.538), and rate of complication (*P *= 0.344). These data suggested that the two groups shared similar severity of injury and clinical outcomes.

### Influence of COVID-19 virus nucleic acid testing normalization on the outcomes of replanted digits with different levels of severity

DASH scores of replanted digits with different levels of severity were significantly improved in both groups (Table 2 and Table 3). Although COVID-19 virus nucleic acid testing normalization significantly prolonged TVS (3.83 ± 0.94 h in group N, 1.77 ± 0.67 h in group C, 95% CI: −2.4 to −1.8, *P *< 0.05), no significant difference of impDASH was observed in patients with same level of severity in both groups (Table 4).

**Table 2:** Comparison of prognosis of emergently replanted finger with different severity before COVID-19 virus nucleic acid testing normalization.

HISS scoring	Preoperative DASH	Postoperative 3 months DASH	*t* value	*P* value
Minor [`*x* ± *s* (%)]	—	—	—	—
Moderate [`*x* ± *s* (%)]	40.6 ± 11.3	7.3 ± 6.5	14.99	<0.0001*
Severe [`*x* ± *s* (%)]	47.4 ± 13.2	6.7 ± 4.8	12.78	<0.0001*
Major [`*x* ± *s* (%)]	70.2 ± 10.7	23.0 ± 14.2	18.44	<0.0001*

COVID-19, corona virus disease 2019; HISS, hand injury severity scoring; DASH, disability of arm-shoulder-hand.

*P value <0.05.

**Table 3:** Comparison of prognosis of emergently replanted finger with different severity after COVID-19 virus nucleic acid testing normalization.

HISS scoring	Preoperative DASH	Postoperative 3 months DASH	*t* value	*P* value
Minor [`*x* ± *s* (%)]	—	—	—	—
Moderate [`*x* ± *s* (%)]	38.1 ± 10.9	9.9 ± 10.4	9.521	<0.0001*
Severe [`*x* ± *s* (%)]	52.6 ± 11.2	12.0 ± 8.1	20.91	<0.0001*
Major [`*x* ± *s* (%)]	69.5 ± 8.4	18.0 ± 6.2	31.92	<0.0001*

COVID-19, corona virus disease 2019; HISS, hand injury severity scoring; DASH, disability of arm-shoulder-hand.

*P value <0.05.

**Table 4:** Comparison of improvement of DASH score of emergently replanted finger with different severity before and after COVID-19 virus nucleic acid testing normalization.

HISS scoring	Before virus nucleic acid testing normalization DASH augment (2021, 6–7)	After virus nucleic acid testing normalization DASH augment (2021, 8–9)	*t* value	*P* value
Minor [`*x* ± *s* (%)]	—	—	—	—
Moderate [`*x* ± *s* (%)]	83.2 ± 13.3	76.0 ± 22.6	1.125	0.269
Severe [`*x* ± *s* (%)]	85.7 ± 10.5	78.5 ± 10.5	1.986	0.055
Major [`*x* ± *s* (%)]	68.1 ± 16.7	74.3 ± 7.8	1.472	0.149

COVID-19, corona virus disease 2019; HISS, hand injury severity scoring; DASH, disability of arm-shoulder-hand.

### Typical case

**Figure 3:**
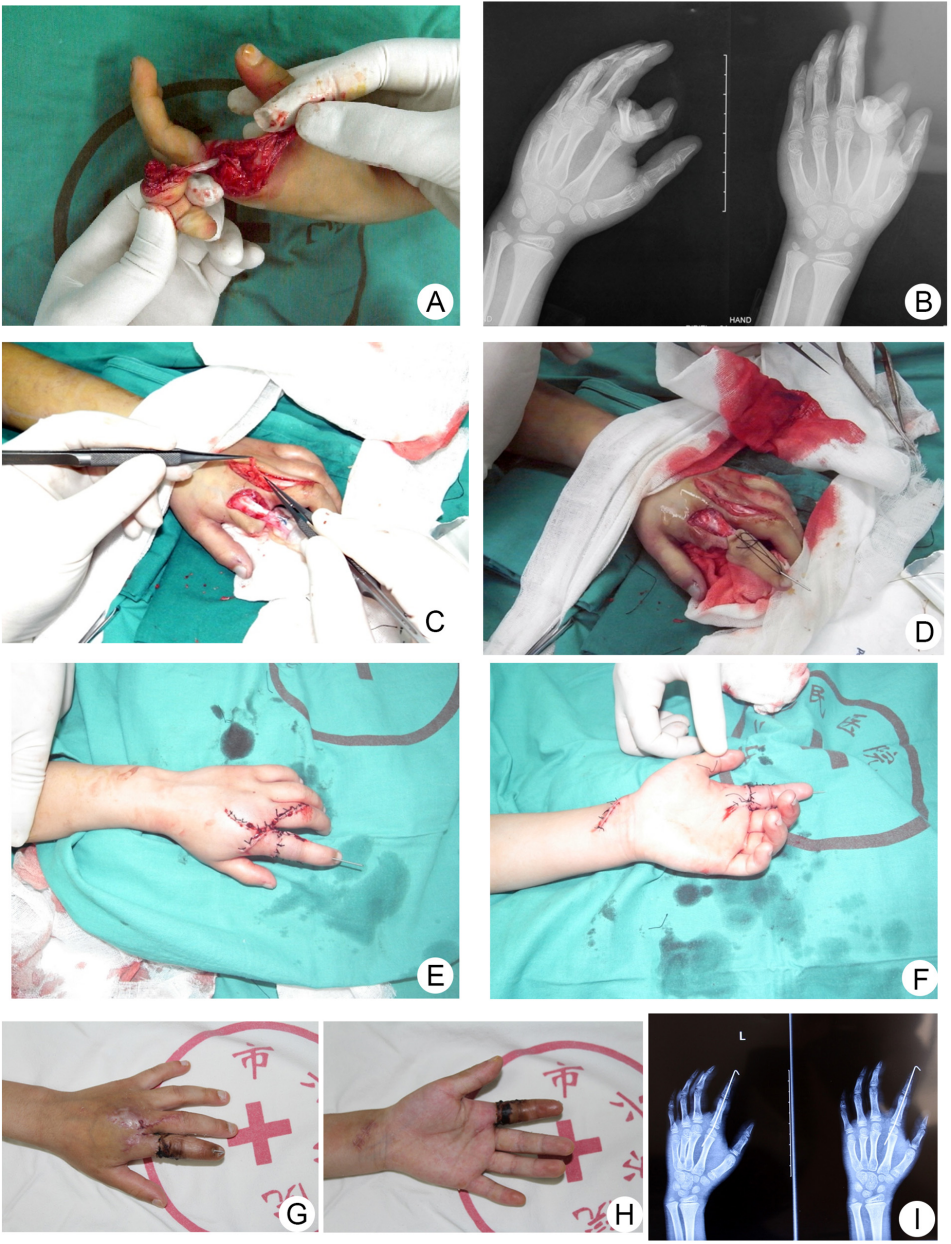
Emergency finger replantation surgery after COVID-19 virus nucleic acid testing normalization. (**A**,**B**) Preoperative appearance and radiography. (**C–F**) Intraoperative appearance. (**G–I**) Appearance and radiography 1 month after surgery. COVID-19, corona virus disease 2019.

After COVID-19 virus nucleic acid testing normalization, a 7-year-old girl visited the orthopedic emergency room 2 h after injury due to incomplete left index finger avulsion. Physical examination reported that the index finger was lacerated at the level of proximal metaphysis of the proximal phalangeal bone with only intact flexor digitorum profundus tendon. HISS scored 38 points and preoperative DASH scored 47.5 according to the physical evaluation (Figures 3A,B). After finishing the preoperative examinations as well as COVID-19 virus nucleic acid test, emergency digit replantation was performed under brachial plexus anesthesia 6 h after injury. Proper palmar arteries of her left index finger were damaged and showed severe spasm lesion; hence, anastomosis was performed between adjacent dorsal digit arteries and distal ends of the proper palmar arteries of the left index finger (Figures 3C–F). The affected finger successfully survived with an impDASH of 82.5% and a DASH score of 8.3 points 3 months after operation (Figures 3G–I).

## Discussion

Several interesting findings of this study are that significantly prolonged time interval from emergency visit to surgery to obtain the result of COVID-19 virus nucleic acid test did not change (1) the finger survival rate, (2) the overall upper extremity function improvement and complication rate compared with patients whose result of COVID-19 virus nucleic acid test was not acquired before operation in group C, and (3) the upper extremity function improvement and complication rate in subgroups with the same severity level graded according to HISS. Although our data do not show the difference in survival rate, it needs to be noticed that the necrotic finger had a higher HISS score before when compared with that after this policy. Moreover, with clear evidence of functional improvement after replantation in both groups, it could be certified that following the policy of obtaining the results of COVID-19 virus nucleic acid test before operation had little impact on the survival of the severed digit as well as on the short-term reconstruction of functionality.

Typically, when facing public health emergency, results of scientific research pave the way for effective policy-addressing while policies become priority disciplines for management social behaviors (18). However, unlike other countries and regions globally, most policies in China are not established with reference sources, which increase the difficulties in daily administration (2). Thus, the dynamics between science and policy often requires postestablishment verification (19). It is obvious that the policy of COVID-19 virus nucleic acid testing normalization has shocked the conventional emergency, surgical, and other medical practices because of prolonged preoperative waiting (6). Patients under life-threatening emergent circumstances, such as acute myocardial infarction and strangulated intestinal obstruction, cannot bear such a long wait, while patients with other emergent situations, including ruptured intracranial aneurysm, were reported to tolerate the wait and exhibit similar outcomes as patients without the wait for result of virus nuclei acid detection (20–23). However, there remains little literature discussing the influence of the policy of COVID-19 virus nucleic acid testing normalization on emergency digit replantation. During our clinical practice, we kept witnessing that patients often complained about the prolonged preoperative wait, wondering if the delay might exacerbate outcomes of the replantation, while patients and surgeons also shared an antipathy to the policy because the new protocol interfered regular working shift, arrangement of surgery, and hospitalization. Patients may also develop negative emotional status during this period, including anxiety and depression, resulting in strained relationships between doctors and patients (24). Hopefully, this study provided clinical evidence for both doctors and patients that waiting for the result of preoperative severe acute respiratory syndrome coronavirus 2 (SARS-COV-2) detection would not change the endings of the replanted digits, since there was no significant difference in overall and leveled functional recovery as well as complication rate before and after COVID-19 virus nucleic acid testing normalization.

Obtaining explicit result of COVID-19 virus nucleic acid test preoperatively after the testing normalization can generally avoid potential SARS-COV-2-infected patients entering emergency operating rooms and postoperative care wards, which will decrease the infection transmission rate within the hospital. However, because of the insufferable pain and serious loss of hand function after finger amputation injury, additional preoperative COVID-19 screening tests, which sometimes enhance the complexity toward emergency healthcare, may result in conflicts between doctors and patients who have a strong will to receive surgical management as soon as possible (25, 26). On the other hand, performing microsurgeries without an explicit detection of COVID-19 requires indiscriminate standard intraoperative third-level protection, according to which a surgeon must wear multiple layers of sterile protection suits under the surgical gown, at least three pairs of gloves, and a mask between the face and the microscope. These sophisticated protection measures may negatively influence the management and efficacy of the digit amputation injury due to difficulties in the wear; blurred vision caused by refraction, reflection, and fogging of the face shield; a decrease in meticulous operation ability caused by the tightness of layers of gloves; and descending communication efficacy among the surgeons, eventually resulting in poor outcomes after replantation surgery (14). Thus, a balance among optimized emergency surgery outcomes, COVID-19 detection, and transmission prevention and control must be established during waves of pandemics. This study revealed that COVID-19 virus nucleic acid testing normalization had little impact on the short-term outcomes of upper extremity function after emergency digit replantation surgery in digit amputation injury cases, even if waiting for the result of COVID-19 virus nucleic acid test as well as the expert group assessment significantly prolonged TVS, which might result in the rise of preoperative anxiety and depression of the injured ones. It is reported that mental status had an impact on the success rate of digit replantation, and preoperative anxiety and depression often brought about unsatisfactory outcomes due to increased inflammation and coagulation process, although the exact mechanism needs further investigation and may be influenced by other factors (27–29). Pessimistic psychiatric changes can also be lured by the compulsory policy of COVID-19 status assessment before emergency replantation surgery, which definitely results in delayed surgical intervention, but no significant difference was found in overall outcomes, indicated by impDASH, and severity level-related outcomes between the two groups (30). The explanation of the phenomenon we found in this study may lie in (1) digits exhibit more tolerance against ischemia because of little capacity of muscles (12, 25); (2) sufficient notification, comfort, and care started from emergency visit and during the wait for examination results; and (3) protection equipment against intraoperative virus transmission has limited restrictions on the operation of microsurgeons when performing replantation surgery for non-SDPs under standard second-level protection, which requires only one layer of gloves and needs no face shields (31).

Limitations of this study are as follows. Because this is a single-centered study, the sample size of the study is rather small; moreover, some of the patients with clear indications for replantation refused the chance to replant the severed finger because of the risk of revision amputation and financial issues. Only short-term outcomes (3 months after replantation) were collected and reported in this study, which indicates that further investigation is needed for long-term influence of COVID-19 virus nucleic acid testing normalization on emergency digit replantation. In addition, no COVID-19 diagnosed case was observed during the sample collection period; hence, the efficacy and necessity of this policy as well as related perisurgical administration schemes for severed digit replantation require further verification. After all, challenges and obstacles faced by microsurgeons after COVID-19 virus nucleic acid testing normalization policy may also be experienced in other fields that have close connection with global public health. Whether the delay, before emergency surgeries until the result of COVID-19 virus nucleic acid test is obtained, can be tolerated or not should be considered and discussed carefully according to indications with humanistic care spirit.

## Conclusion

Despite the preoperative delay, the policy of COVID-19 nucleic acid testing normalization does not have explicit influence on the short-term outcomes of emergency digit replantation surgery. With this evidence, microsurgeons could pay attention to the patients’ anxiety and spend more effort in comforting them during the prolonged preoperative wait. These insights may also have implications for other emergency department resource management whenever a social crisis occurs.

## Data Availability

The data that support the findings of this study are available on request from the corresponding author. The data are not publicly available due to privacy or ethical restrictions. Requests to access the datasets should be directed to the corresponding author.
